# Supplementary material for: SpiR is a gut microbial enzyme that drives cholesterol conversion
Source: Nat Commun. 2026 Apr 14;17:3495. doi: 10.1038/s41467-026-70820-6 (PMC13079761; doi:10.1038/s41467-026-70820-6)
Supplement: Supplementary file 1 — Supplementary Information [file 41467_2026_70820_MOESM1_ESM.pdf]

Supplementary Information

SpiR is a gut microbial enzyme that drives cholesterol conversion

Authors:

Gabriela Arp[1,†], Sophia Levy[1,†], Angela K. Jiang[1,2], Keith Dufault-Thompson[2], Aoshu Zhong[3], Maggie Grant[1], Yue Li[4], Xiaofang Jiang[2,*], Brantley Hall[1,4,*]

Affiliations:

[1]Department of Cell Biology and Molecular Genetics, University of Maryland, College Park, College Park, Maryland, USA

[2]National Library of Medicine, National Institutes of Health, Bethesda, Maryland, USA

[3]Division of Molecular and Cellular Biology, Eunice Kennedy Shriver National Institute of Child Health and Human Development, National Institutes of Health, Bethesda, Maryland, USA

[4]Center for Bioinformatics and Computational Biology, University of Maryland, College Park, College Park, Maryland, USA

[†]These authors contributed equally

[*]Corresponding authors: xiaofang.jiang@nih.gov, brantley@umd.edu

Supplementary Information includes:

- Supplementary Figures 1–8

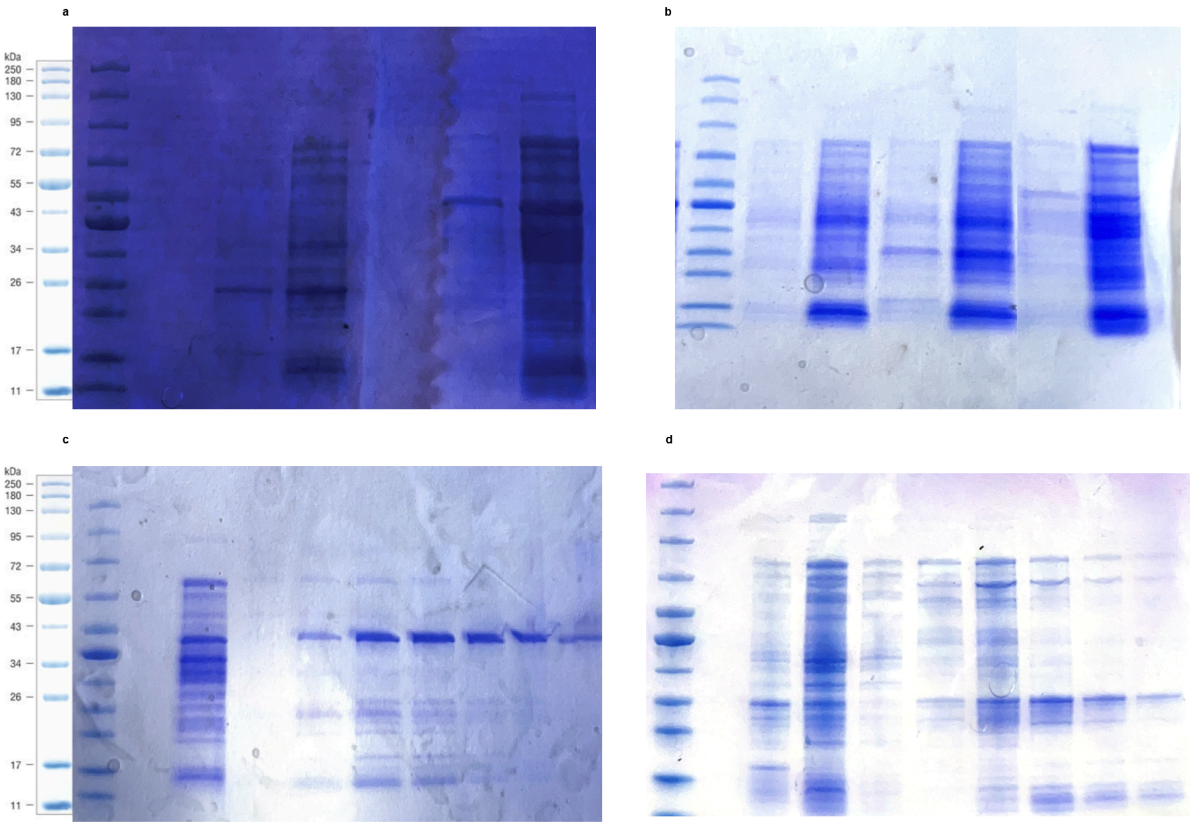

Figure S1: SpiR and IsmA expression levels monitored by SDS-PAGE band intensity. A broad range of blue pre-stained protein markers (11-250 kDa) was used for protein size determination. **(a)** Lanes from left to right: IsmA-uninduced, IsmA-induced, IsmA supernatant, SpiR-uninduced, SpiR-induced, and SpiR supernatant. The gel demonstrated a strong band at ~33 kDa in ismA samples and ~72 kDa in SpiR samples that were not present in the vector control (not shown). **(b)** Lanes from left to right: vector control-induced, vector control supernatant, IsmA-induced, IsmA supernatant, SpiR-induced, and SpiR supernatant. The gel demonstrated an ~33 kDa band for ismA and a ~72 kDa band for SpiR, which were not seen in the vector control lanes. **(c)** SpiR purification from left to right shows post lysis supernatant, elution fraction 1, 2, 3, 4, 5, 6. This purification was performed on a HisTrap Excel column. **(d)** IsmA purification from left to right shows post lysis pellet, post lysis supernatant, column flowthrough, elution fraction 1, 2, 3, 4, 5. This purification was performed on a HisTrap Excel column.

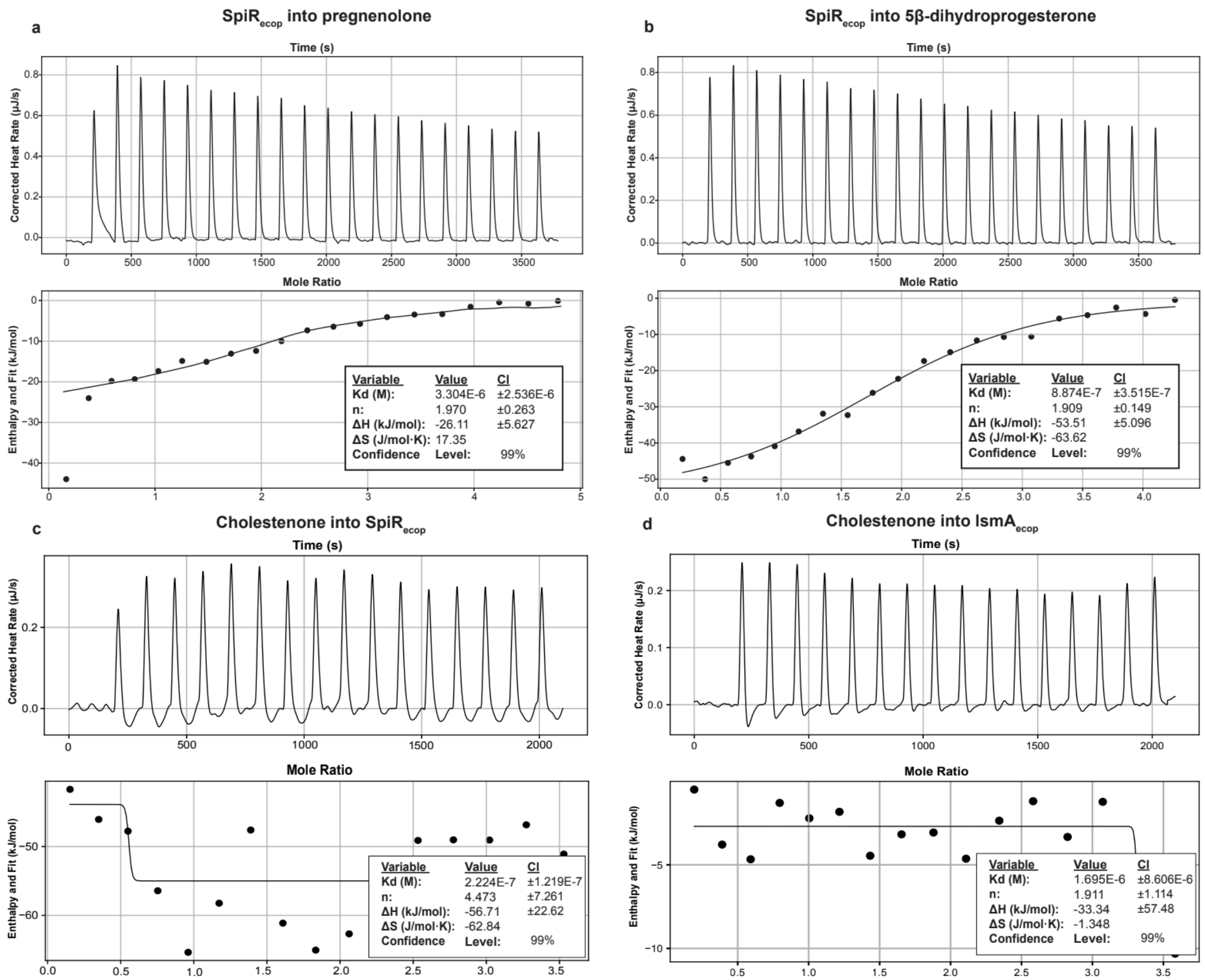

Figure S2: SpiR binding to steroid hormones. Binding of SpiR to **(a)** pregnenolone and binding of SpiR to **(b)** 5β-dihydroprogesterone measured by ITC. Heats of binding are shown in the upper panel and integrated binding isotherms fitted with the independent binding model are shown in the lower panel. For both experimental conditions, the ligand was in the calorimeter cell and 2.5 µL injections of the recombinant SpiR were injected. The binding of 5β-dihydroprogesterone to SpiR was investigated using a 5 µm ligand and 50 µm protein, and pregnenolone to SpiR was studied using a 15 µm ligand and 150 µm protein. The binding between cholestenone and **(c)** SpiR and cholestenone and **(d)** IsmA measured by ITC. For both experimental conditions, the recombinant proteins were in the syringe and 3.1 µL of ligand were injected. The binding of cholestenone to SpiR and IsmA was investigated using 50 µm ligand and 5 µm protein. The thermodynamic parameters with standard errors were calculated based on each curve using the NanoAnalyze Software package.

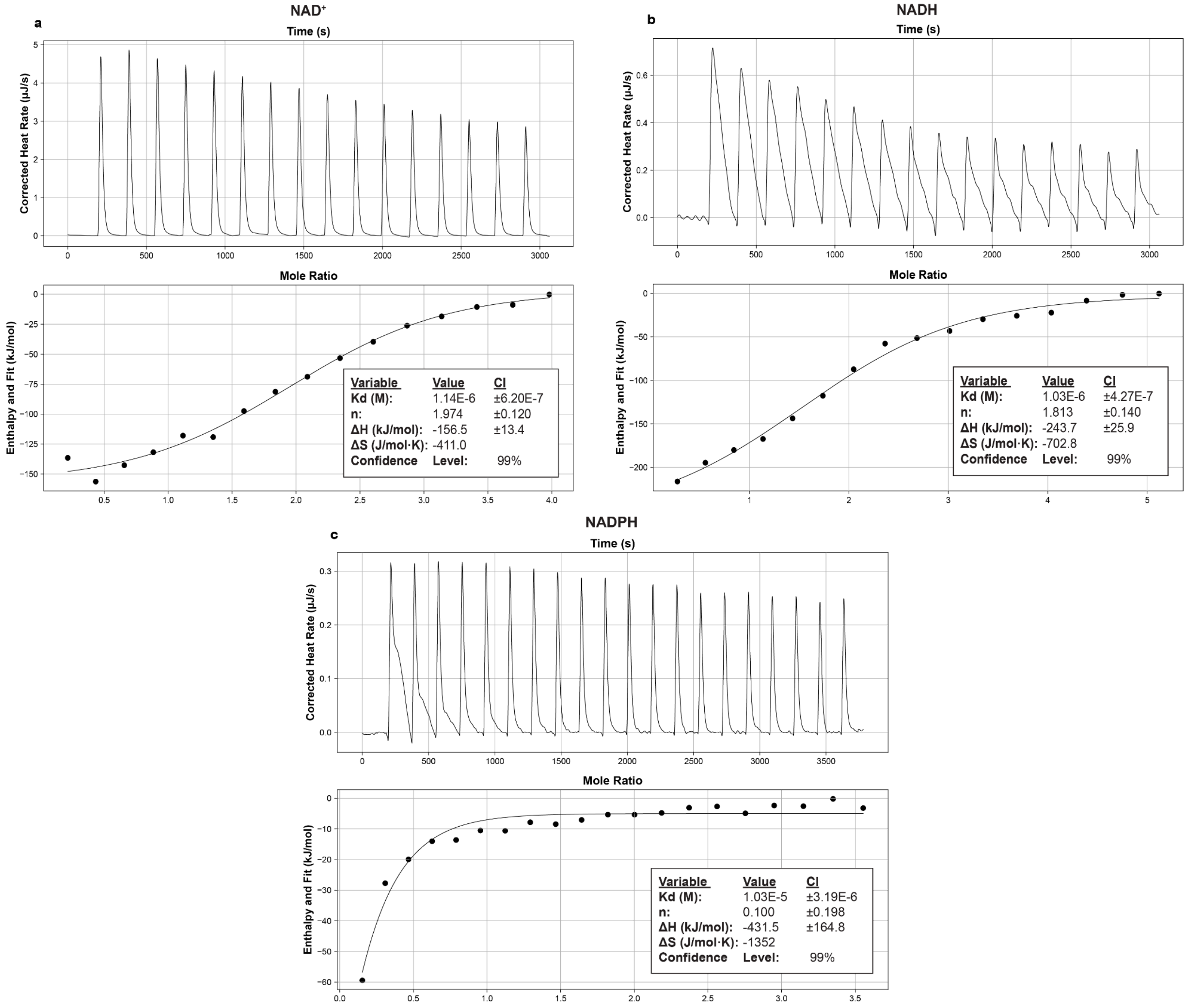

Figure S3: Characterization of SpiR cofactor binding. (a) NADH titration into SpiR using a 50 µm ligand and 5 µm protein, **(b)** NAD^+ titration into SpiR using 100 µm ligand and 10 µm protein, **(c)** NADPH titration into SpiR 100 µm ligand and 10 µm protein were all measured by a standard ITC at 25°C. The raw heats of binding are shown in the upper graph of each panel, whereas the integrated binding isotherms are shown in the lower panel. Independent binding model fitting was used for the data integration and analysis. The thermodynamic parameters with standard errors were calculated based on each curve using the NanoAnalyze Software package.

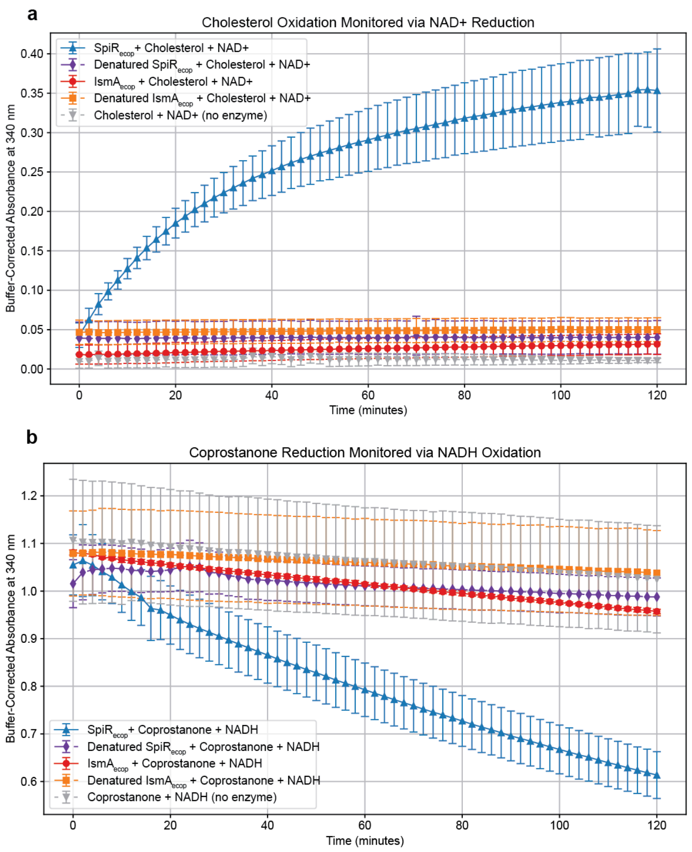

Figure S4: Establishing biological activity assay using NADH and NAD^+ oxidation.: SpiR and IsmA activity was measured by tracking the **(a)** oxidation of cholesterol to cholestenone and **(b)** reduction of coprostanone to coprostanol. This was done using a kinetic spectrophotometric method that monitored the reduction of NAD^+ at 340 nm and oxidation of NADH at 340 nm over a time span of two hours. The standard reaction mixture consisted of 0.1M sodium phosphate buffer at pH 6.5, 50 µM hormone, 400 µM NADH or NAD^+, and 4 µM enzyme. Each experimental condition was performed in triplicate. Vertical lines are shown at each collected time point to represent the variability in OD_{600} values across the biological replicates (n = 3) within a single experimental condition.

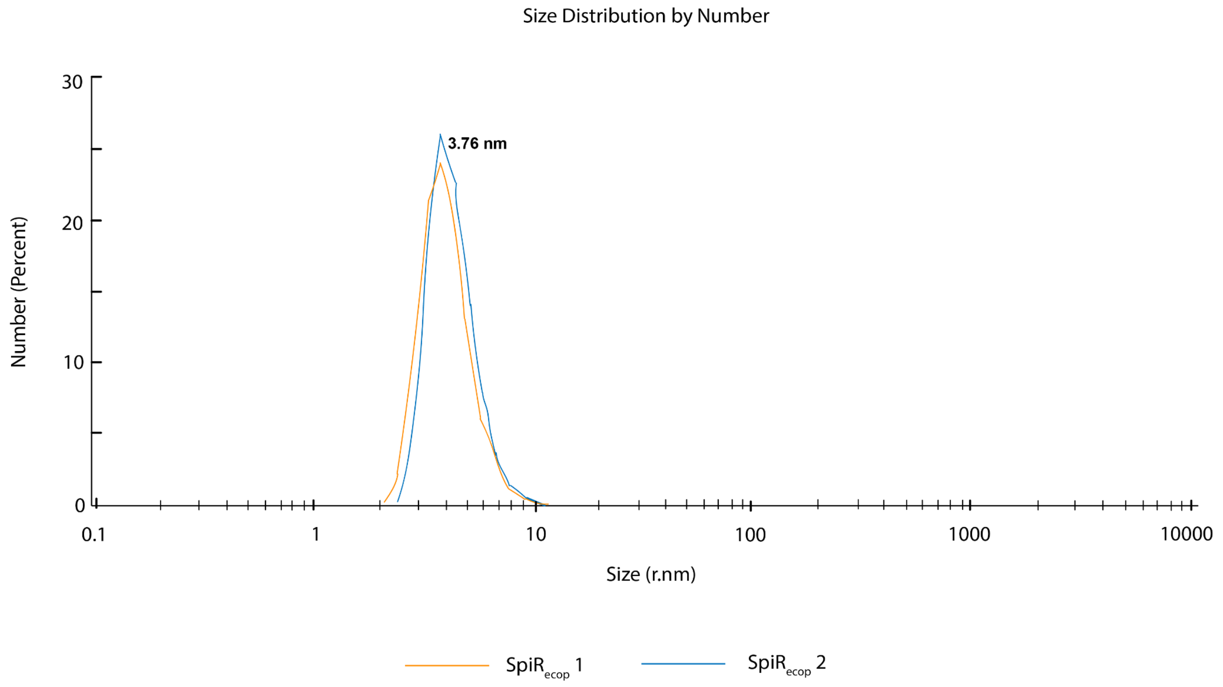

Figure S5: Oligomeric status of SpiR assessed by dynamic light scattering (DLS). The hydrodynamic radius is reported in nanometers and indicates that SpiR is primarily present as a 3.76 nm dimer. The size distribution has been reported by numbers to correct for overrepresentation of the large aggregates in intensity-based measurements, which are present in low abundance but dominate light scattering.

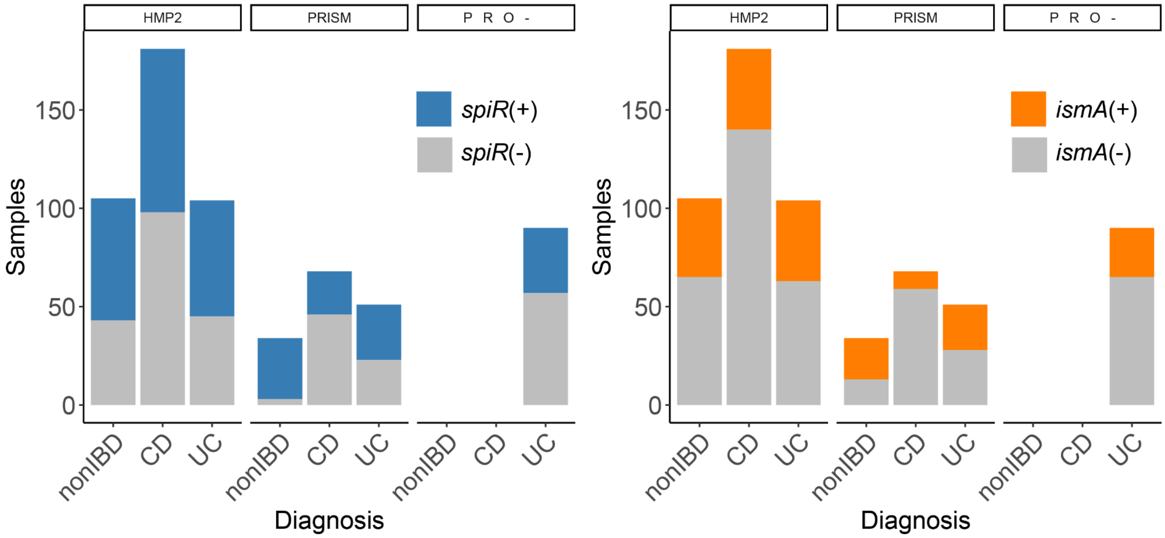

Figure S6: *spiR* and *ismA* presence across IBD disease states. Relationship between IBD diagnosis and the presence of *spiR* (blue) and *ismA* (orange) in three cohorts (HMP2, PRISM, and PROTECT).

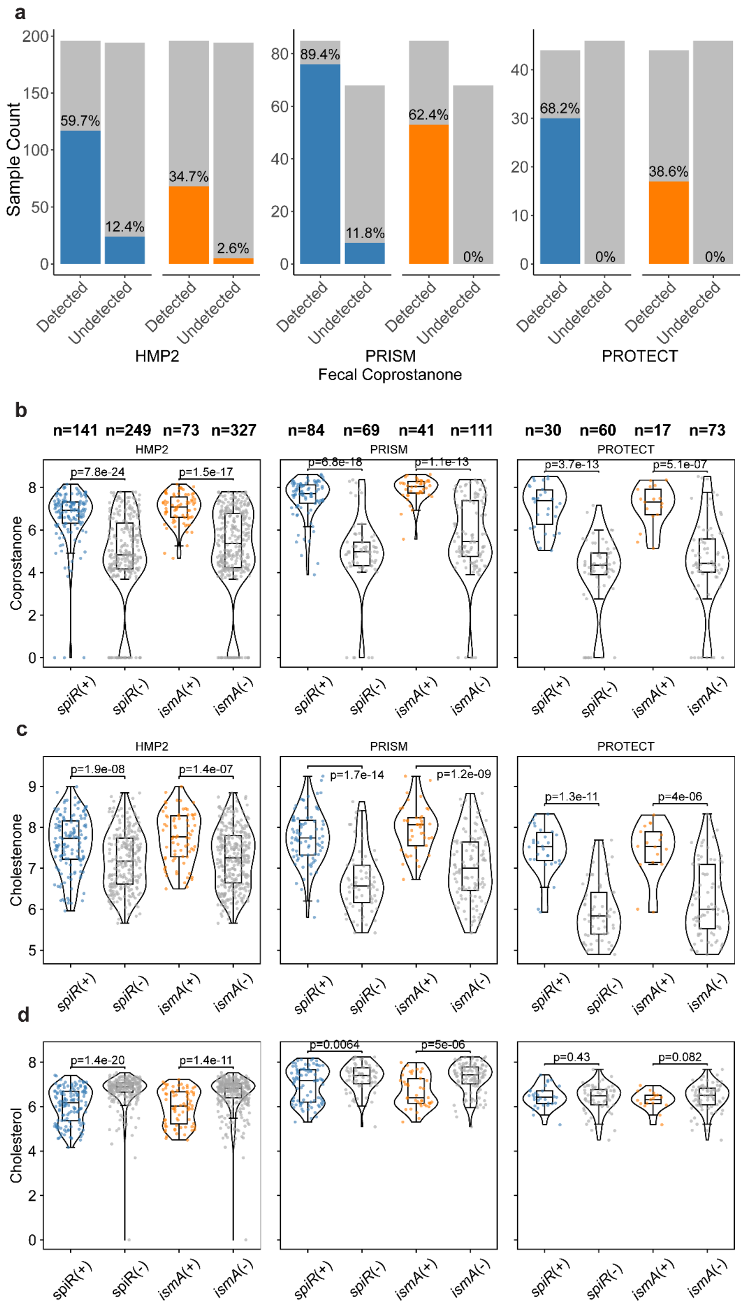

Figure S7: *spiR* and *ismA* presence in coprostanol producers and non-producers. (a) The presence of *spiR*-encoding species (blue) and *ismA*-encoding species (orange) in relation to whether corprostanone was detected in the stool metabolomics data. Violin and box plots showing the relationship between the presence of *spiR* encoders, *ismA* encoders, and log10 peak area of metabolites (**(b)** coprostanone, **(c)** cholestenone, **(d)** cholesterol). *P*-values were calculated using the Mann-Whitney U test between encoders and non-encoders for each study and metabolite.

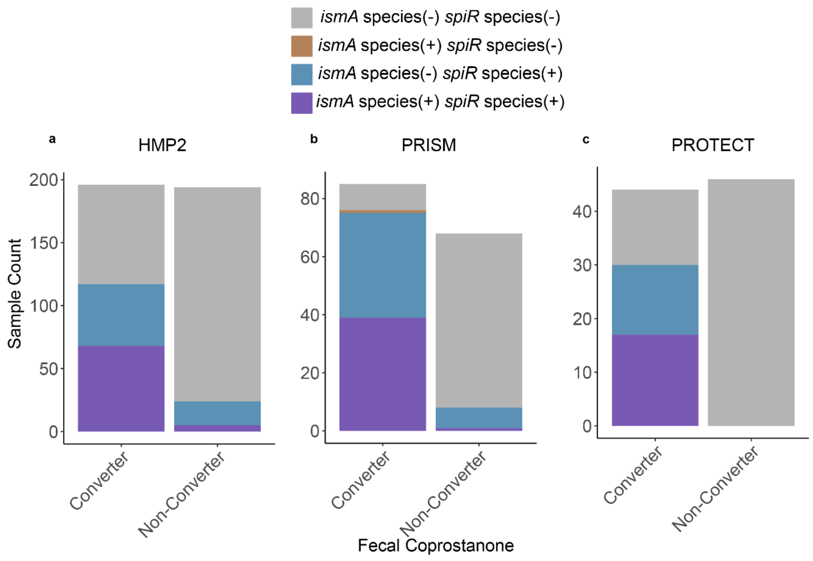

Figure S8: Species presence of *spiR*- and *ismA-encoding* species in gut metagenomes. Stacked bar plots show the distribution of species classified by the presence of *ismA* and *spiR* genes in fecal metagenomes from converters and non-converters across the HMP2 (a), PRISM (b), and PROTECT (c) cohorts. Species were grouped into four categories: those lacking both genes (*ismA–*/*spiR–*, gray), those carrying only *ismA* (*ismA+*/*spiR–*, brown), those carrying only *spiR* (*ismA–*/*spiR+*, blue), and those carrying both genes (*ismA+*/*spiR+*, purple).
