# Supplementary material for: SpiR is a gut microbial enzyme that drives cholesterol conversion
Source: Nat Commun. 2026 Apr 14;17:3495. doi: 10.1038/s41467-026-70820-6 (PMC13079761; doi:10.1038/s41467-026-70820-6)
Supplement: Supplementary file 2 — Description of Additional Supplementary Files [file 41467_2026_70820_MOESM2_ESM.pdf]

File Name: Supplementary Data 1

Description: Supplementary Data 1 contains information about hydroxysteroid dehydrogenase (HSDH) sequences used for phylogenetic analysis.

File Name: Supplementary Data 2

Description: Supplementary Data 2 contains *ismA*/*spiR* homolog sequences and their accession numbers and taxonomic distribution.

File Name: Supplementary Data 3

Description: Supplementary Data 3 contains the accession numbers and taxonomy information of all strains of *ismA* and *spiR* encoders.
